# The Role of Students’ Self-Regulated Learning, Grit, and Resilience in Second Language Learning

**DOI:** 10.3389/fpsyg.2021.800488

**Published:** 2021-12-22

**Authors:** Liang Wang

**Affiliations:** Beijing Language and Culture University, Beijing, China

**Keywords:** second language students, grit, higher education, resilience, self-regulated learning

## Abstract

It has been established that grit has a fundamental role in the learning and teaching process since gritty learners are more likely to take part in classroom activities and they are also motivated to deal with challenges in difficult circumstances. In addition, to guard against these hardships as well as self-control in responding to unpredicted circumstances, a similar construct arouses in positive psychology called resilience that describes perseverance and emphasizes people’s abilities. Besides, language learners’ engagement and performance in the foreign or second language classroom can be improved through self-regulated learning (SRL) which is viewed as one of the most inspected issues in learning and psychology. A present review has been conducted to scrutinize the relationship between language learners’ SRL and learners’ resilience and grit based on their positive theoretical relationship with educational success. Consequently, the issue of educator training and administrative training is illuminated through several aspects.

## Introduction

For years, educational centers and academic establishments around the globe have been worried about the abandonment rates of their learners (Brown, 2012). Indeed, education is one of the major factors for societal development and social advancement and in any community; academic failure and boredom are a significant concern (Keegan, 2017; Wibrowski et al., 2017). Nonetheless, there has been a growing extent of the literature on positive feelings in language learning (Dewaele and MacIntyre, 2014). Indeed, there have been numerous elements that have affected the success of learners over time, such as skill, effort, planning, and self-confidence and in every corner of the globe, educators strive to help students succeed and achieve (Sedlacek, 2017). There are many reasons why some learners excel more than others, which can be difficult for educators to determine so they are primarily concerned with improving methods that facilitate students’ learning (Mohan and Kaur, 2021). Researchers shift their efforts away from psychological attributes to focus on non-cognitive attributes, which results in successful discoveries. Numerous data support the notion that non-cognitive features affect educational performance (Steinmayr et al., 2019). In addition to psychological factors, other factors influence the behavior and development of college learners (Sedlacek, 2017). Two non-cognitive factors, namely, grit, and resilience, can affect students’ accomplishment and achievement (Wolters and Hussain, 2015).

Success is not only determined by the capability of the learner but also depends on the interaction of intellectual skills and individual attributes, like grit, plus a strong desire to achieve (Bazelais et al., 2016). A combination of talent and effort results in success, as claimed by Duckworth et al. (2007). In line with Sedlacek (2017), characteristics or traits associated with the inspiration and adaptation of college learners are the non-cognitive elements. Two non-cognitive aspects that affect the success and wellbeing of students are grit and resilience which directly affect learners’ achievement and success (Strayhorn, 2014; Wolters and Hussain, 2015). Resilience and grit are both crucial elements of organizational success, and they are connected to flexible, effective, and enthusiastic teams (Bartz, 2017). Social skills, appearance, physical fitness, and cognitive capacity are far less useful predictors of success than resilience and grit (Duckworth, 2016). To improve their learners’ academic performance and success, many educators are now encouraging resilience and grit and young people and older individuals who face difficulties will be more likely to persist in their academic goals if they have resilience and intellectual motivation (Cassidy, 2015).

As an adaptive strategy, resilience refers to the act or achievement of making effective adaptations to challenging situations or threats (Martin and Marsh, 2008). In simple terms, resilience is the capability to handle or adjust to adverse or difficult conditions, whether expected or actual (Chisholm-Burns et al., 2019). As with many skills other than cognitive functioning, resilience is not necessarily inherited but rather developed by learners through various circumstances and managed feelings (Trigueros et al., 2020). The construct of resilience is universally used and designated in a variety of ways by many people; it presents a multidimensional concept that is applicable in several different contexts. People who have achieved success often possess the trait of resilience (Mohan and Kaur, 2021). As O’Connor et al. (2014) stated, a person’s resilience is a function of the experiences they have in the course of their life rather than a fixed characteristic developed in some areas at a given age. Learners can learn by being resilient while seeking advanced learning or careers. Being competent despite unfavorable circumstances is known as resilience and has to do with the capability to recover from psychological damage (Civita, 2000). As a result, resilience is an outcome of the interaction between people, their social contexts, and their cultural contexts; it is significantly correlated with psychological wellbeing measurements (Lavin Venegas et al., 2019; MacIntyre et al., 2019; Xue, 2021).

Essentially, Duckworth (2016) stated that grit comes from being resilient in the face of obstacles or difficulty, which is the aspect of being gritty. As a component of constructive psychology, grit is defined as the tendency to persevere over long periods as an indicator of long-term achievement. To make learners motivated and more gritty, supportive psychology was created to help them recognize and realize their capabilities which now focus on studying positive aspects of people, such as optimism, creativity, and happiness/contentment (MacIntyre et al., 2019; Wang et al., 2021). Grit is characterized as the display of passion and persistence when pursuing a long-term objective (Duckworth et al., 2007; Areepattamannil and Khine, 2018). People who have a high degree of grit are more likely to be involved with their work than other people in which gritty means laying out plans and pursuing them for a long time and continuing to move forward regardless of difficulties and also gritty entails how you cope with difficult circumstances, similar to resilience (Suzuki et al., 2015). Resilience and grit are expected to protect learners against adverse outcomes. As declared by Duckworth (2016), resilience refers to the capability to overcome major challenges while grit, contrary to resilience, is the ability to deal with daily obstacles, setbacks, and challenges (Perkins-Gough, 2013). Additionally, a learner with grit achieves their own goals in life, whereas someone with resilience might not have specific goals (Tang et al., 2020). A growing number of studies have shown that grit can significantly impact educational, scholastic, and career success (Bazelais et al., 2016). Grit can be described as the motivation and commitment to achieve a goal over time. It has been linked with low levels of depression signs and adverse effects, as well as high levels of wellbeing and good feelings (Datu et al., 2019). Thus, grit has been perceived as a person’s solid feature or disposition, like conventional character traits, that affects his/her assertiveness and practice throughout various circumstances (Duckworth and Quinn, 2009). As opposed to those with lower levels of grit, people with a great amount of grit are predicted to display more perseverance when chasing their objectives despite failures, interruptions, or different types of interference. Within academic frameworks, grit is displayed as a possible crucial impact on results like students’ commitment, engagement retention, involvement, success, and the possibility of graduation (Strayhorn, 2014; Xie and Derakhshan, 2021).

Moreover, as far as self-regulated learning (SRL) measures are concerned, grit’s persistence of efforts predicted all types of SRL measures; however, continuous engagement did not forecast such outcomes (Wolters and Hussain, 2015). It turns out that grit, which is not intellectual, is contained of two components, including constancy of effort and continuity of desire (Duckworth et al., 2007). Continuity of desire is characterized by the capability to stick with one purpose so the learners remain focused on the objectives that have been established at the beginning and do not quickly become distracted (Duckworth, 2016). Persistence of effort refers to learners’ persistent attempts in the case of difficulties to complete the tasks and accomplish their desired outcomes (Zimmerman, 2002).

In addition, the learners need to be motivated enough to control their cognitive processes, performance, and behavioral responses to the classroom demands. To enhance motivation, cognitive processes, psychological wellbeing, and behavioral control, individuals must be self-regulators (Duru et al., 2014). Indeed, the other factor related to learning success in the academic context is SRL that refers to the comprehensive process of education that consists of intellectual, psychological, social, emotional, and personal elements (Panadero, 2017; Müller and Seufert, 2018). Studies have shown that SRL is important for learners’ educational progress since it makes them more motivated and academically successful (Anam and Stracke, 2016). Self-regulated teaching is a flexible educational approach that focuses on the learner creating his or her reactions, feelings, and skills and learning toward attaining his or her personal goals (Zimmerman and Schunk, 2011; Zhang and Zhang, 2019). In recent years, education has been recognized as one of the most important tasks for instructors to carry out: instruct learners how to become independent, responsible individuals who can directly regulate their education (Šteh and Šarić, 2020). SRL is an alternative significant issue in educational achievement which refers to the capability of the human personality to achieve their education forms (Ormrod et al., 2016). The concept of SRL has become a crucial element for comprehending, assessing, and enhancing learners’ educational achievement in recent decades (Zimmerman and Schunk, 2011). Self-regulated learners tend to be intellectually, emotionally, and psychologically vigorous and dynamic in their education, and those learners have a greater likelihood of having successful professional careers in both conventional and non-conventional fields (Wibrowski et al., 2017). Wolters and Hussain (2015) indicated that SRL partly facilitates the connection between persistence and educational presentation. The thoughts, emotions, and practices that learners utilize at some stage in education are explained by the SRL process. It consists of the individual elements which have formerly been related to instructional resilience, like self-efficacy, setting of objectives, inspirational beliefs, and self-control (Freeman et al., 2004; Han and Wang, 2021). Motivating factors are often considered an essential element in SRL, and a student’s effort, persistence, and ability to adapt are commonly recognized traits (Zimmerman, 2002).

Furthermore, Almeida (2016) integrated the objective element of grit with self-regulated educational techniques and he noted that the inclination for long-term objectives encourages learners to utilize self-regulated educational techniques. In the same vein, Wolters and Hussain (2015) proposed that self-regulated educational techniques can be regarded as mediating elements between the objective aspect of grit and academic success. These techniques involve personal objective choice, overseeing and regulating cognition, inspiration, and practice during the educational experience to eventually achieve personal objectives (Wolters and Hussain, 2015; Hrbackova and Suchankova, 2016). The theory of self-regulated education offers a viewpoint that emphasizes the implicit inherent inspirational indicator and the explicit objective element of grit.

A recent common discussion about the notion that developing grit might improve learning outcomes has been supported by the obvious conclusion that educational achievement is the result of the combination of effort and sustained interest, especially in challenging circumstances (Huang and Zhu, 2017). Consistent with multiple findings, there is a significant interaction between grit and qualities of self-regulation like intelligence (Wolters and Hussain, 2015) and self-awareness (Arslan et al., 2013). A study carried out by Strayhorn (2014) proved a progressive connection between grit and academic achievement in African American college learners at principally white organizations. It was found in another study carried out by Duckworth and Quinn (2009) that grit is an indicator of a career change. People with greater points of grit are less likely to change careers than people with lower levels of grit. This result could indicate that college learners who have grit are less expected to change or switch majors. Muenks et al. (2017) elucidated Duckworth et al.’s (2007) conceptualization of “consistency of interest” as more activity- and objective-directed, embracing long-term practice as opposed to reflecting a personal inclination toward a specific theme (individual attentiveness) or a personal interest stimulated by a certain circumstance (situational attentiveness). Even though the relationship between SRL and academic achievement is clearly shown, not much research has been conducted to date based on the researcher’s knowledge on the role of SRL on learners’ resilience and grit. The present review addresses the gap by investigating whether grit and resilience together can be used to apprehend students’ SRL.

## Review of Literature

The main constructs of the study are defined as follow:

### Grit

Grit is one of the non-cognitive attributes including perseverance, self-reliance, motivation, consciousness, and goal setting, as well as a strong non-cognitive aspect (Credé, 2018). Individuals who have grit are passionate about reaching their long-term objectives, resilient when they fail, and persistent in their efforts to accomplish them (Von Culin et al., 2014). Grit involves two scopes, namely, perseverance of effort (PE) and consistency of interest, in which the former leads to success despite setbacks, while the latter fosters intentional practice to achieve goals (Credé et al., 2016). Along with Duckworth (2016), grit as a cognitive trait has been predicted to be a tool for accomplishment in a wide variety of fields and it has attracted significant interest among scholars. Moreover, persistence was originally conceived as a character strength while it is being called by Seligman (2002) as grit that has been extended, elaborated, popularized, and established by Duckworth et al. (2007) as the capability to persist in a focused effort to accomplish a task despite the obstacles that may appear is tied to grit (Sturman and Zappala-Piemme, 2017). Likewise, Strayhorn (2014), who studied grit and its influence on academic performance in learners, found that learners who were gritty were more likely to perform well on exams than learners who were less gritty. Learners who are grittier also tend to be more hopeful about education and have higher levels of education (Eskreis-Winkler et al., 2014). As stated by Keegan (2017), grit has been suggested to take on a key function in supporting learners to develop their English language proficiency in spite of the problems they deal with. In all facets of life, social expression is accomplished through grit as a fundamental and crucial topic that can be considered an ethical value or ability (Lee, 2020). It is revealed that people with greater degrees of grit achieve higher grades, including those in college, and continue to learn at more innovative stages than those with inferior degrees of grit, while the degree of grit determines participation in extracurricular activities and higher school grades (Duckworth et al., 2007; Duckworth and Quinn, 2009).

### Resilience

Resilience is related to defensive and weak elements inside and outside a person which affects a person’s adjustment to changes and traumatic experiences that bring about a lack of homeostasis (Brewer et al., 2019). International learners are recommended to possess resilience so they can cope with the challenges of their new setting and adapt to it and a resilient learner is defined according to his or her ability to tackle change. As a result, resilience is concerned with how learners can recover after a challenging situation or deal with it (Portnoy et al., 2018). Resilience is a process that concentrates on the association between learners and their setting and measures the relationships between various aspects of mental wellbeing and academic achievement (Van der Meulen et al., 2020). In addition to having the capability to adapt to difficult circumstances, resilience can be understood as the power to adapt to changes in circumstances (Southwick et al., 2014). In other words, resilience is the aptitude to react confidently to adversity or hardship in a given situation (Masten and Reed, 2002). A resilient learner develops the ability to cope better with errors and hardships rather than allow negative situations to hamper their performance. Educational success seems to require particular cognitive capabilities. Having resilience means being able to survive and deal with the adverse influences of stressful circumstances and various challenges encountered daily (Richardson, 2002). In the teaching career, resilience is a vivacious fundamental to comprehend instructing and educational approaches that happen while people associate their assets with topic-oriented ones and make use of operative approaches and tactics to triumph over difficulties and sustain their wellbeing (Greenier et al., 2021; Xue, 2021).

### Self-Regulated Learning

Self-regulated learning describes how one regulates his or her emotions, activities, and context while studying or achieving a goal (Butler et al., 2017). The SRL method is complicated for the following reasons: psychological, behavioral, and external factors (De Boer et al., 2013; Wang and Guan, 2020). In pursuit of individual objectives, SRL is a dynamic, continuous process of individuals setting learning goals (Zimmerman and Schunk, 2011) and utilizing multiple controls and monitoring strategies to manage cognitive abilities, interest, attitude, and environmental factors to attain those goals (Zusho, 2017). Learners who perform self-regulation are those who are active in and aware of their educational development. It is more likely for them to persevere and to achieve better results when they are faced with challenging academic settings (Zusho, 2017; Wang and Guan, 2020). Students utilize various educational techniques that would help them learn and apply the material. Therefore, the process by which students actively assume responsibility for and control over their education is known as SRL. Students using SRL have a clear understanding of why and how a specific self-regulation strategy is utilized. Pintrich (2000) characterized SRL as a productive and active cycle in which students establish objectives for their education and then try to oversee, control, and adjust their inspiration, awareness, and practice, directed and inhibited by their objectives and the appropriate characteristics in the setting. An individual has an SRL strategy when he or she organizes, perceives, and adjusts their activities according to a purpose (Zimmerman, 2002). Self-regulation involves more than merely acquiring skills, since it implies an awareness of one’s development, enabling one to make adjustments in behaviors and attitudes to become a better learner and apply to learn more efficiently (Colthorpe et al., 2019). The manner, in which people control their actions, as well as their beliefs, is known as self-regulation (Renninger and Hidi, 2016). In essence, self-regulated education is the cycle through which learners take over their education. Learners direct the inspirational, intellectual, and behavioral dimensions of their education by including different sub-cycles, namely, objective setting, progress tracking, activation of related past knowledge, participation, and regulation of educational techniques and assessment (Zhang and Zhang, 2019; Teng and Zhang, 2021). Self-regulated students possess a strong determination and perseverance in their education and positive inherent inspiration is one of the main indicators of the self-regulated educational process (Hrbackova and Suchankova, 2016; Wang and Guan, 2020). If learners are not inherently inspired to learn, they may not be engrossed in using their energy to develop their education or themselves. SRL is designed as a dynamic and adaptable cycle that includes three vast but overlapping aspects, namely, use of strategy, inspiration, and metacognition (Butler et al., 2017; Cleary, 2018). The use of strategy, which is the first aspect, refers to the actions and approaches that learners utilize to improve education and/or to control and regulate their feelings, practices, feelings, and educational settings (Dunlosky et al., 2013). The second dimension, inspiration, mirrors the cycle by which people begin and maintain practices like endeavor, perseverance, and choice. Showing inspired practices is regularly a function of inspirational beliefs, including objective orientation, self-efficacy, task advantages, and attitude (Cleary, 2018). Metacognition, which is the last aspect, talks about the level of consciousness and control of an individual over his/her thought; in other words, how to plan, monitor, and assess your thoughts while learning (Zimmerman, 2002).

## Implications and Future Directions

Constructed on this review of the literature, it is demonstrated that language learners’ SRL has a major impact on higher education and has positive effects in Second Language Learning. Learners can learn self-regulation regardless of their educational background through SRL and those learners who report academic success use SRL strategies to increase their chances of success in educational settings, as found in Zimmerman’s research. The upshots gleaned from this review of the literature indicated that SRL is highly significant in instruction since it advances learners’ cognitive awareness and strategic management and it helps with education by orienting learners’ inspiration and endeavors toward significant objectives, all of which could enhance academic achievement. Learners who implement SRL successfully utilize educational techniques, evaluate their progress, and persevere in carrying out objective-oriented practices. Similarly, research demonstrates that learners get better outcomes in their class when they utilize SRL techniques, by which they set academic objectives and when they utilize techniques to attain such objectives. SRL is a method of self-discovery and a way of learning that is exciting. It allows learners to determine what is best for them based on their abilities and learning preferences. Having planned their target goal according to their educational background and abilities, students feel more confident about their skills. Self-regulated students demonstrate a high degree of autonomy in education and are talented at controlling and supervising inspirational, intellectual, and behavioral dimensions of the learning cycle (Zimmerman, 2002). It was proposed that the personality attributes of people constantly affect the method knowledge is processed, thereby creating specific learning practices. According to the specificity of the objective, an individual’s devotion to the objective and their task-relevant information leads to an emphasis on objective-related activities, self-regulation, perseverance, and the utilization of task-relevant information and techniques (Locke and Latham, 2002).

Developing personal motivation when tackling a new educational task can help learners enhance their attitudes about learning. Therefore, language learners who display resilience and believe they can complete an assignment are more likely to apply SRL techniques to boost and enhance their learning experience. In the classroom, learners who use self-regulated methods often exhibit inherent motivation and have the ability to learn independently. Behavioral and mental methods are always used by such learners, as well as self-regulatory learning, which is always centered on understanding when, how, and what to do (Wibrowski et al., 2017). Moreover, learners who rely on their capability of carrying out a task are more likely to persist in completing it than learners who do not believe they are capable. It has become clear over the past years that every person, even youths during childhood and adolescence, confronts hardships in life and that trauma is not an immediate result of an occurrence but how a person deals with it. Thus, it is required of all those involved to attend to, nurture, and educate youths to endeavor toward resilience as an asset for growth or as a group of regular coping abilities for every learner. By doing so, joy and wellbeing can be increased, and the difficulties, fluctuations, and challenges confronted in life can be better managed (Portnoy et al., 2018). Resilient learners maintain high degrees of success and achievement despite stressful occurrences and circumstances. A multi-faceted construct, resilience is one of the most significant individual elements which influence educational achievement and is the competence of facing the most difficult problems, bearing, and managing the most catastrophic occurrences, complex circumstances, and drawbacks (Oke et al., 2016).

In addition, some features like grit can result in persistent self-regulation and assist learners to accomplish objectives and goals during different situations (Eskreis-Winkler et al., 2014). A gritty person is likely to give more effort to endure stressful and long tasks to perform better and grit is also connected to emotional control. Also, gritty individuals can better utilize their assets in the long run by coordinating their efforts and actions toward achieving their targets (Eskreis-Winkler et al., 2014). Gritty learners can deal with more complex and difficult situations due to their objective-oriented tendency and their ability to regulate their emotional state (Ceschi et al., 2016). Along with self-regulated training, gritty learners engage more in challenging and demanding situation (Wolters and Hussain, 2015). Further, Eskreis-Winkler et al. (2014) reported greater feelings of wellbeing and less likelihood to quit as grittier learners exhibited meaningfully more self-regulated mechanisms of learning, including valuing the material they have learned, building their self-efficacy, using smart and motivational learning techniques, and helped them delay less as well (Wolters and Hussain, 2015).

The importance of understanding and promoting self-regulation in classrooms requires educators to be familiar with factors influencing a student’s talent to self-regulate and the methods they can use to identify and promote SRL by learners that activates their level of resilience in case of challenges. Learners can discover how to manage their emotions, feelings, and reactions by using SRL. Gritty learners can set short- and long-term objectives for their education, make a plan to achieve them, act as a motivator, and maintain their concentration on their objectives and success. Educators who teach learners self-regulation are more successful at fostering educational success, engagement, and continuous learning. By exposing learners to new developments in SRL that stimulate new interests and motivations, syllabus designers and material developers should focus on designing classroom activities that engage encourage and stimulate learners to be accountable for their learning. Additionally, more experimental studies with a mixed-methods study containing quantitative data associated with grit, resilience, and SRL, together with qualitative data collection procedures, can be carried out to permit for investigation which provides an expanded standpoint of the topic and diminish any predispositions.

## Author Contributions

LW conceptualized, collected data, analyzed data, and drafted the manuscript to submit it to Frontiers in Psychology.

## Funding

This research project is supported by Science Foundation of Beijing Language and Culture University (supported by “The Fundamental Research Funds for the Central Universities”) (Approval number 21YJ210004).

## Conflict of Interest

The author declares that the research was conducted in the absence of any commercial or financial relationships that could be construed as a potential conflict of interest.

## Publisher’s Note

All claims expressed in this article are solely those of the authors and do not necessarily represent those of their affiliated organizations, or those of the publisher, the editors and the reviewers. Any product that may be evaluated in this article, or claim that may be made by its manufacturer, is not guaranteed or endorsed by the publisher.
